# Efficient induction of spawning of Northern leopard frogs *(Lithobates pipiens)* during and outside the natural breeding season

**DOI:** 10.1186/1477-7827-11-14

**Published:** 2013-02-25

**Authors:** Vance L Trudeau, Frederick W Schueler, Laia Navarro-Martin, Christine K Hamilton, Elizabeth Bulaeva, Amanda Bennett, William Fletcher, Lisa Taylor

**Affiliations:** 1 Centre for Advanced Research in Environmental Genomics, Department of Biology, University of Ottawa, K1N 6N5, Ottawa, Ontario, Canada; 2 Bishops Mills Natural History Centre, K0G 1T0, Bishops Mills, Ontario, Canada; 3 Department of Biology, Trent University, K9J 7B8, Peterborough, Ontario, Canada; 4 Method Development and Applications Unit, Biological Assessment & Standardization Section, Environment Canada, 335 River Road, K1A 0H3, Ottawa, Ontario, Canada

**Keywords:** *Lithobates pipiens*, Induced spawning, Leopard frogs, Hormone

## Abstract

**Background:**

Amphibian declines are now recognized globally. It is also well known that many anurans do not reproduce easily in captivity, especially when held over long periods, or if they require hibernation before breeding. A simple method to induce spawning and subsequent development of large numbers of healthy tadpoles is therefore required to meet research and conservation goals.

**Methods:**

The method is based on simultaneous injection of both female and male leopard frogs*, Lithobates pipiens* (formerly called *Rana pipiens)* with a cocktail of a gonadotropin-releasing hormone agonist (GnRH-A) and a dopamine antagonist. We call this the AMPHIPLEX method, which is derived from the combination of the words amphibian and amplexus. Following injection, the animals are thereby induced, and perform amplexus and natural fertilization under captive conditions.

**Results:**

We tested combinations of a GnRH agonist with 2 different dopamine antagonists in *L. pipiens* in the breeding season. The combination of des-Gly^10^, D-Ala^6^, Pro-NHEt^9^-GnRH (0.4 micrograms/g body weight; GnRH-A) with metoclopramide hydrochloride (10 micrograms/g body weight; MET) or domperidone (DOM) were equally effective, producing 89% and 88% successful spawning, respectively. This yielded more than 44,000 eggs for the 16/18 females that ovulated in the GnRH-A+MET group, and more than 39,000 eggs for the 15/17 females that ovulated in the GnRH-A+DOM group. We further tested the GnRH-A+MET in frogs collected in the wild in late autumn and hibernated for a short period under laboratory conditions, and report a low spawning success (43%). However, GnRH-A priming 24 hours prior to injections of the GnRH-A+MET cocktail in animals hibernated for 5–6 weeks produced out-of-season spawning (89%) and fertilization (85%) comparable to those we observed for in-season spawning. Assessment of age and weight at metamorphosis indicated that *L. pipiens* tadpoles resulting from out-of-season spawning grew normally and metamorphosed successfully.

**Conclusion:**

We provide evidence for successful captive breeding of the leopard frog, *L. pipiens*. This simple protocol can be used to obtain large numbers of eggs in a predictable, timed manner.

## Background

Global amphibian loss is considered a major factor in the contemporary "sixth mass extinction" according to Wake and Vreedenberg [1]. The potential detrimental ecological impacts have been discussed and debated extensively [1-5]. Perhaps 30% of known amphibians are endangered. Significantly, it was Nace [6] more than 40 years ago, who recognized that some populations of *Lithobates pipiens* (formerly *Rana pipiens)* in the U.S.A. were already declining in the mid-1960s. Today, Western North American populations, including those in Alberta, British Columbia, Colorado, and Nevada have dramatically declined [7-10]. Northeastern Ontario populations of *L. pipiens* have also been declining over the last 40 years [11].

Nace and colleagues [6,12] were probably amongst the earliest to suggest the need for captive breeding and eventual domestication of frogs. Timed breeding in captivity is therefore a critical step for the propagation of any threatened or endangered species. To address this challenge, Nace et al. established the University of Michigan Amphibian Facility in the late 1960s [6,12]. They reported [6,12] that spawning in captivity was possible but involved injections of pituitary extracts, which necessitates sacrifice of adult leopard frogs, thus defeating the main purpose of a captive colony of a species in decline. The costs, risks, inefficiencies and inappropriateness of injection of pituitary extracts have been discussed by Clulow et al. [13]. Unfortunately, the early attempts [6,12] at induced breeding did not lead to establishment of a spawning method for captive leopard frogs. This was at a time before the discovery of the hypothalamic decapeptide gonadotropin-releasing hormone (GnRH), and little was known about the neuronendocrine control of reproduction in frogs.

While it is now known that GnRH and GnRH agonist treatments can stimulate pituitary luteinizing hormone (LH) release in frogs [14,15], they may not effectively induce spawning without co-treatments with other hormones or neuroactive agents [16-18]. This is indicative of the existence of an inhibitory neuroendocrine mechanism controlling the surge release of LH required for ovulation and spawning. Until recently, this possibility had not been considered important in frog reproduction despite clear but limited evidence to the contrary. Electrolytic lesions in the hypothalamus and infundibular regions of the hibernating frog *Rana temporaria*[19] increased GnRH and LH release and advanced spawning times, thereby establishing the existence of an LH-inhibitory system in the frog neuroendocrine brain. It is known that these brain areas contain the catecholamine dopamine (DA) [20] and DA type 2 receptors have been found in the frog pituitary [21,22]. Immunocytochemical visualization of DA neuronal fibres in the hypothalamus and median eminence of *Rana ridibunda* indicates that DA can be delivered to the pituitary [23]. The DA agonist bromocriptine can inhibit LH release and ovulation in *R. temporaria* in some situations [24]. Moreover, long-term implantation of silastic pellets containing the DA antagonist metoclopromide (MET) induced ovulation in hibernating *R. temporaria*[24]. These data indicate that DA is an important inhibitor of LH release in frogs as it is in numerous fish species, birds and some mammals, including sheep and humans [25]. Browne et al. [18] explored the effects of combinations of hormones on spawning in *Bufo fowlerii*. In that study, they used the DA antagonist pimozide and concluded that pimozide may increase spawning in some situations and hormone combinations. However, pimozide is not specific to DA receptors, and acts on adrenergic and serotoninergic receptors in addition to DA receptors [26], so it’s use should be avoided. Nevertheless, together these studies led us to test several dopamine antagonists in *L. pipiens*.

In our first report on hormonal induction of spawning in *L. pipiens,* it was clear that the combination of des-Gly^10^, D-Ala^6^, Pro-NHEt^9^-GnRH (GnRH-A) and the specfic DA D2-receptor antagonist MET gave the best results. We named the approach the AMPHIPLEX method, a term that derives from the combination of the words amphibian and amplexus. Amplexus refers to the specific reproductive behaviour of frogs where the male grasps the female, helping to stimulate ovulation and after some delay fertilizes the eggs as they are laid. In one year in the breeding season, we obtained fertilized egg masses from 100% of females, while in the second year this was only 60% [27]. We considered these in-season spawning results for *L. pipiens* a good first step [27]. On the other hand, out-of-season breeding success was very low in *L. pipiens*[27], indicating that significant improvements are needed.

Here we report on the efficient, large-scale spawning induction during the reproductive season of *L. pipiens* following co-injection of GnRH-A and either of the DA antagonists MET or domperidone. Given the efficacy and ease of use of the GnRH-A+MET combination, we also successfully induced breeding out-of season and obtained thousands of viable tadpoles. In our case, the main reason for this planned breeding is to obtain healthy tadpoles in a timed manner for physiological, ecotoxicological and epidemiological studies [28,29]. In the long-term our goal is to establish captive colonies so that harvesting of wild eggs can be stopped because the iconic North American frog *L. pipiens* is unfortunately in decline in several regions of the traditional range [7-12].

## Methods

### Experiment 1: Large-scale induction of spawning in the spring breeding season of *L. pipiens*

Mature leopard frogs (51 females, 81 males) were collected near Bishops Mills, Ontario during their spring migration to breeding pools between April 9 and 13, 2011. The animals were kept cold (4°C) temporarily and were transported on April 19 (water temperature 6-7°C) to the National Wildlife Research Centre, Ottawa and housed outside in large 380 L high-density polyethylene (Rubbermaid) tanks containing 200 L of water as described previously [27]. The sexes were housed separately and acclimated to the tanks until injection. Typically, 2 mature females and 3 mature males were placed in each tank with submerged branches as spawning substrate. Mean (± SD) body weights for all females and males used in this experiment were respectively 51.1 ± 7.6 and 29.5 ± 6.2 g. Each breeding tank was only observed once per day at the time when water temperatures were recorded in the morning. Water temperature in the tanks varied between 12-16°C at the time of injection (Day 1) and respectively 13-17°C, 11-12°C, and 9-11°C on Day 2, 3 and 4. Thereafter, tank temperatures were 15-16°C.

Animals were injected between 12:00–17:00 h on April 30, 2011. Animals in the control group (16 females and 27 males) were injected intraperitoneally (i.p.) with saline (0.7% NaCl; 1 μl/g) and DMSO (1 μl/g) vehicles using a 26-gauge needle attached to a disposable 1-ml syringe. The AMPHIPLEX method is based on injections of a mixture of des-Gly^10^, D-Ala^6^, Pro-NHEt^9^-GnRH (Bachem H4070; 0.4 μg/g body weight (BW); GnRH-A) and metoclopramide (Sigma M0763; 10 μg/g BW; MET) as reported previously [27] except that both GnRH-A and MET were dissolved together in saline. These animals were also injected with DMSO control (1 μl/g) to be comparable to the other groups. There were 18 females and 27 males in the GnRH-A+MET treatment group. We also wanted to test another dopamine antagonist, therefore, a third group was treated with GnRH-A (0.4 μg/g body weight; GnRH-A) and domperidone (generous gift from Janssen Pharmaceutica; 10 μg/g BW; DOM). It was necessary to dissolve DOM in DMSO because it is not soluble in water. There were 17 females and 27 males in the GnRH-A+ DOM group.

On Day 5 post-injection, the resulting egg masses were weighed and placed in individual aquaria at room temperature (21°C) to observe and record embryonic development. Post-spawning body weight of females was also recorded. Additionally, 3 small subsamples of the egg masses were weighed and the number of eggs counted under a dissection microscope. The average number of eggs per gram of subsample was multiplied by total egg mass weight to estimate total fecundity. Relative fecundity was estimated by dividing the total number of eggs by the pre-spawning body weight of females. Percent fertilization rates were estimated by determining the proportion of developing and non-developing embryos 4 days following spawning.

It should be noted that the females that did not spawn were sacrificed and it was determined that they did have eggs.

### Experiment 2: Out of season induction of spawning of *L. pipiens* in captivity

For the breeding trials that occurred outside the normal spring breeding season, mature leopard frogs were collected near Bishops Mills, Ontario from mid November-early December 2011 using submerged minnow traps in a deep pond, which is a traditional, successful hibernation site for leopard frogs in this region. The animals were kept cold (4°C) temporarily and were transported and housed in-doors in tanks at 4°C at the University of Ottawa aquatic facility. Mean (± SD) body weights for all females and males used in this experiment were respectively 51.9 ± 8.5 and 25.5 ± 6.6 g. Two trials were conducted.

In the first trial, 7 females and 7 males were placed separately in breeding tanks (internal diameter 0.9 m, depth 0.36 m, total volume of 230 litres) and maintained at 4-6°C, in the dark for 23 days. Thereafter they were exposed to 6h light (14:00–20:00h; 100W Phillips Natural Light bulb, 1200 lumens) for one day, then 14h light (06:00–20:00h for the remainder of the experimental period. The temperature was gradually increased to 16-17°C over 6 days, starting on the same day that lights were turned on. The following day, animals were injected i.p. in the afternoon with GnRH-A+MET. Four females with 4 males were placed in one tank, and 3 females with 3 males were placed in another tank.

For the second trial, we set out to improve spawning success using a priming pre-treatment with a low dose of GnRH-A before injection with GnRH-A+MET. Twelve females and 16 males were kept separately in holding tanks (internal diameter is about 0.67 m, depth about 0.33 m, total volume of 115 l) and maintained at 4-6°C, in the dark for 5–8 weeks. Thereafter, they were exposed to the same photoperiod/temperature regimen described above for Trial 1. For the priming pre-treatment, females and males were injected i.p. between 14:00–14:30 with a low dose of GnRH-A (0.04 mg/g; Day 1). Animals were moved to the breeding tanks but the sexes were kept separated. Twenty-four hours later (Day 2), animals were injected i.p. with GnRH-A+MET. Immediately following this injection, 3 females and 4 males were placed in a given breeding tank (16-17°C).

For both trials in Experiment 2, the resulting egg masses were weighed and the post-spawning body weight recorded. Fertilization and fecundity measurements followed the protocol described for Experiment 1.

The females that did not spawn were sacrificed and it was determined that they did have eggs.

### Experiment 3: Development and survival of tadpoles generated from out-of-season breedings

Three egg masses obtained from pairs injected only with GnRH-A+MET (masses 1–3) and 2 egg masses from the GnRH-A priming protocol (masses 4–5) were further assessed. These 5 egg masses were studied to determine tadpole survival, growth, and metamorphic rates in addition to potential genetic (parental) influences on these parameters. Five fertilized egg masses were removed from the breeding tanks from the out-of-season breeding trials reported above and placed individually in 20 L glass tanks at room temperature until hatching and development to Gosner Stage (Gs) 25. [30]. Percent fertilization estimates for these eggs masses varied between 10 and 92.7%. The tadpoles were reared in a Tecniplast-ZebTec recirculating system, starting with addition of Gs25 tadpoles. The water was maintained at a constant temperature (21°C), pH (7.4), and conductivity (~1000 μS). Tadpoles were initially stocked at a density of approximately 2 tadpoles/L of water (e.g., 15 tadpoles per 8L ZebTec tank), and this was not adjusted as the tadpoles developed. There were 5 replicate tanks for each egg mass. Tadpoles were fed commercial rabbit pellets ad libidum. Excess food was removed daily.

At approximately Gs40, a floating Styrofoam plate and plastic grill was placed in each tank so that tadpoles could rest, and prepare to leave the water at metamorphosis. To reduce the chance of drowning, upon reaching Gs45 froglets were transferred to 100 × 15 mm glass Petri dishes with 5 ml water. Once metamorphosis was completed (Gs46, complete tail regression), animals were anaesthetised in tricaine-methanesulfate (MS-222, Sigma) and sacrificed.

For each metamorph, the snout-vent length (SVL; precision, 0.1mm), weight (W; precision, 0.1mg), type and incidence of deformities, and total days post-fertilization to complete metamorphosis (DTM) were recorded. The condition factor (k) was calculated using SVL and weight measurements as follows: k = (W(g)/SVL(cm^3^)*100. Survival at Gs45 was calculated by comparing the number of tadpoles reaching that stage with the total number of Gs25 embryos at the beginning of the experiment. The percentage of Gs25 embryos that completed metamorphosis (Gs46) was also determined.

### Statistical analyses

Online freeware was used for statistical analysis of spawning induction data. Fisher’s Exact test (http://www.graphpad.com) and Student’s T-test (http://studentsttest.com) were used. Data are reported as mean ± SD.

For the data on tadpole development, growth and survival, the statistical analyses were performed using SPSS version 15.0 (Chicago, Illnois, USA). Normality was verified using Shapiro–Wilks’ test and logarithmic transformations were performed to ensure normality when required. Homoscedasticity of variances was verified with Levene’s test. A one-way analysis of variance (ANOVA) was used to test for statistical differences between egg masses in SVL, W, K and DTM. Differences in percentage of animals that complete metamorphosis, incidence of malformations, survival to Gs45 and death rate between Gs45 and Gs46 were determined using the non-parametric test Krustal-Wallis. Data are expressed as mean ± SD. In all tests, differences were accepted as significant when p < 0.05.

All procedures followed the animal care guidelines of the University of Ottawa and the Canadian Council on Animal Care.

## Results

### Experiment 1: Large-scale induction of spawning in the spring breeding season of *L. pipiens*

One pair of saline-injected control animals was observed in amplexus on Day 3 This couple remained in amplexus for 2 days but did not spawn.

In the treated groups, the first egg masses were seen on Day 3. On Day 3, 5/18 pairs were in amplexus and 2 egg masses were laid in the GnRH-A+MET group. On Day 3, 9/17 pairs were in amplexus and one egg mass was laid in the GnRH-A+DOM group. By Day 5, spawning activity stopped. Regardless of treatment, all egg masses laid were fertilized (>90%) and led to development of healthy tadpoles. In the GnRH-A+MET group 16/18 (89%) females laid eggs. In the GnRH-A+DOM group 15/17 (88%) females laid eggs (see Table 1). There was no difference in the proportion of females laying eggs in the GnRH-A+MET and GnRH-A+DOM groups (p=1.00, Fisher’s Exact test).

**Table 1:** Spawning success in a large-scale in-season breeding trial in Spring 2011

**Spawning in *L. pipiens***	**Egg masses (%)**	**#eggs/female**	**Relative fecundity (#eggs/g BW)**	**Total # eggs collected**
**Control**	0/16 (0)	0	0	0
**GnRH-A+MET**	16/18 (89)****	2764 ± 690^ns^	54 ± 10^ns^	44,224
**GnRH-A+DOM**	15/17 (88)****	2642 ± 597^ns^	54± 12^ns^	39,630

Data were analysed with Fisher’s Exact Test (egg masses; ****p=0.0001), and T- Test (GnRH-A+MET vs. GnRH-A+DOM; (# eggs per female and relative fecundity; ns, p>0.05). The total number of eggs obtained per treatment was estimated (# females that spawned x #eggs/ female). See Methods for details on experimental protocols.

The body weight loss in females after spawning was on average 20.0% (range 11.3-35.4%) in the GnRH-A+MET group and 20.3% (range 10.2-35.1%) in GnRH-A+DOM group (p>0.05; T-test). Total egg mass weight was also not different (p>0.05; T-test) between the treatment groups and was 137.4 ± 34.5 g for GnRH-A+MET and 130.3 ± 27.2 g for GnRH-A+DOM. Similarly, the total number of eggs per egg mass were not different (p>0.05; T-test) between the treatments and were 2764 ± 690 and 2642 ± 597 for GnRH-A+MET and GnRH-A+DOM, respectively (Table 1). One estimate of fecundity is eggs per gram pre-spawning body weight. This was estimated to be 54 ± 10 and 54 ± 12 for the GnRH-A+MET and GnRH-A+DOM groups, respectively, which were not significantly different (p>0.05). We obtained approximately 44,224 eggs for the 16 females that ovulated in the GnRH-A+MET group. For the GnRH-A+DOM group we obtained approximately 39,630 eggs for the 15 females that ovulated.

### Experiment 2: Induction of spawning of *L. pipiens* in captivity in winter outside of the normal spring breeding season

Data from this experiment is shown in Table 2. In Trial 1 of Experiment 2 without GnRH-A priming, 3/7 (43%) females treated with GnRH-A+MET laid eggs. The first egg mass was laid on Day 2, and the others on Day 4. All 3 egg masses were fertilized (range 10- 81%). At the end of the trial, the females were sacrificed. There were virtually no eggs left in the females that spawned but the 4 others were full of eggs that were not released.

**Table 2:** Spawning success in out-of season breeding trials (Winter 2011–2012)

**Spawning in *L. pipiens***	**Egg masses (%)**	**%Fert**
**Trial 1 -** GnRH-A+MET	3/7 (43)	47 ± 35
**Trial 2 -** GnRH-A priming, GnRH-A+MET	11/12 (92)****	85 ± 15*

Data were analysed with Fisher’s Exact test (Egg Masses; ****p=0.0001) and T- Test (% Fert; percent fertilization, * p=0.046). See Methods for details on the 2 trials.

In Trial 2 of Experiment 2 with GnRH-A priming (Table 2), 11/12 (92%) females treated with GnRH-A+MET laid eggs, which was a significant improvement in spawning rate compared to Trial 1 (p=0.0001; Fisher’s Exact test). On Day 3, which was 1 day after injection with GnRH-A+MET, 5/12 couples were in amplexus. On day 4, 6 other couples were in amplexus. The first egg masses (4/12) were also observed on Day 4. By Day 5, 11/12 females laid eggs. No other spawning activity was observed after Day 5.

Total egg mass weight was 127.5 ± 55.6 g in Trial 1 and 132.4 ± 59.4g in Trail 2 and was not significantly different (p=0.90; T-test). The total number of eggs per egg mass was 2,052 ± 871 in Trial 1 and 1,584 ± 777 in Trial 2 (p=0.46; T-test). Relative fecundity as indicated by eggs per gram pre-spawning body weight was estimated as 36 ± 15 and 31 ± 14 for Trial 1 and Trial 2, respectively (p=0.69; T-test). We recorded high % fertilization levels in Trial 2 of Experiment 2. On average this was 85% (range 53-98%) and was significantly higher (p=0.046; T-test) than fertilization estimates obtained in Trial 1 (Table 2).

The average body weight for in-season females that laid eggs was 51.1 ± 7.6 g and was not different from the average body weight of 51.9 ± 8.5g for all out-of season females that laid eggs. Similarly, average weight of egg masses per female was also not different (p>0.05; T-test; see Table 3). The total number of eggs laid per female was significantly lower by 43% in the out-of-season spawnings compared to the in-season spawnings (p<0.0005). The average number of eggs per gram body weight was 54 ± 10 for the females from the spring 2011 breeding season treated with GnRH-A+MET. In contrast the average number of eggs per gram body weight for females primed with GnRH-A, then injected with GnRH-A+MET in the out-of-season breeding trial was 31 ± 14, which was 43% lower (p=0.00009; T-test).

**Table 3:** Comparison of fecundity in eggs obtained from the in-season and out-of-season spawnings

**Spawning in *L. pipiems***	**Weight of Egg masses (g)**	**# Eggs/female**	**Relative fecundity (#eggs/g BW)**
**In season**			
(no priming, GnRH-A+MET)	135 ± 6	2744 ± 118	54 ± 10
**Out of season**			
(GnRH-A priming, GnRH-A+MET)	131 ± 15	1584 ± 234***	31 ± 4*

Data were analysed with T- Test (***p<0.0005; *p<0.05). Please see Methods for detailed description of the experiments.

### Experiment 3: Development and survival of tadpoles generated from out-of-season breedings

After hatching, Gs25 tadpoles were raised to metamorphosis. The average time to metamorphosis ranged from 85.0 to 118.8 days, and is significantly different between some of the egg masses (Table 4). There were 5 replicate tanks for each of these 5 egg masses. Overall, the average % of individuals reaching metamorphosis was 74.1 ± 16.5. Individually, egg masses 1, 2, 3 and 4 were very similar with 80.0 ± 13.3, 82.7 ± 13.8, 77.3 ± 11.2 and 81.3 ± 9.9% of animals completing metamorphosis, respectively. Examples of normal tadpoles and metamorphs from the out-of-season breeding are shown in Additional file 1: Figure S1. The dominant malformation in tadpoles was spinal curvature, and the average was 8% for the entire population of tadpoles studied. Most of these were observed for one egg mass. The incidence of abnormalities was 0, 1.4, 3.1, 0 and 35.5 % in egg masses 1, 2, 3, 4 and 5, respectively. Egg mass 5 was significantly different (p < 0.05) from the others, with 49.3 ± 7.6% reaching metamorphosis. It was also egg mass 5 that had the lowest survival, shortest time to metamorphosis, and the highest incidence of malformations. While the higher mortality and abnormalities are likely related to the genetic background of the parents, the bigger size and increased condition factor of the tadpoles from egg mass 5 most probably relates to rearing conditions. We did not adjust tadpole densities during development to adjust for the different rates of mortality. Regardless, it is clear that there are differences between the breeding couples in captivity that reflect typical variations in natural populations.

**Table 4:** Characteristics of metamorphosed froglets from 5 different parental pairs

**Egg mass (%Fert)**	**N (%)**	**DTM**	**W (g)**	**SVL (cm)**	**k**
**1** (47.6)	63 (84%)	105.2 ± 25.3^b^	0.86 ± 0.23^ab^	0.021 ± 0.002^a^	8.9 ± 1.0 ^a^
**2** (10.0)	64 (85%)	118.8 ± 26.1^c^	0.83 ± 0.20^a^	0.021 ± 0.002^a^	8.7 ± 1.0 ^a^
**3** (92.7)	61 (81%)	108.7 ± 21.4^bc^	0.88 ± 0.21^ab^	0.021 ± 0.002^a^	9.3 ± 1.7 ^ab^
**4** (53.5)	62 (83%)	105.0 ± 29.2^b^	1.04 ± 0.26^c^	0.023 ± 0.002^b^	8.6 ± 0.8 ^a^
**5** (88.2)	39 (52%)	85.0 ± 17.9^a^	0.95 ± 0.19^bc^	0.021 ± 0.002^a^	9.6 ± 2.4 ^b^

Data (± SD) for days to complete metamorphosis (DTM), snout-vent length (SVL), weight (W) and condition factor (k) for each egg mass (1–5) is shown. Means that are significantly different are indicated by different letters (p<0.05). Abbreviations: N, total number out of 75 Gosner Stage 25 embryos in the group that survived to metamorphosis (% survival is shown in parentheses), DTM, days to metamorphosis; SVL, snout-vent length; W, weight; k, condition factor; %Fert, percent fertilization.

## Discussion

We report on the efficient hormonal induction of spawning in the Northern leopard frog, *L. pipiens.* This simple protocol can be used to obtain large numbers of eggs for in-season and out-of-season breeding.

In our first study on hormonally-induced spawning in anurans [27] we tested 2 different DA antagonists, pimozide and MET, in combination with several GnRH agonists. From those early results it was clear that MET was more effective than pimozide. In the natural spring breeding season in the current study, none of the control animals laid eggs, consistent with previous observations for *L. pipiens* under the captive conditions in outdoor breeding tanks [27]. In marked contrast, 89 and 88% of female in the GnRH-A+MET and GnRH-A+DOM treatment groups, respectively, laid eggs that were all fertilized by the males. The well-characterized DA D2-type receptor antagonist MET is water-soluble and readily crosses the blood–brain barrier. The site of action of MET to antagonize DA receptors and promote successful spawning may therefore be at both the level of the brain and pituitary. Domperidone is also a well-characterized DA D2 receptor antagonist that is not water-soluble and does not cross the blood–brain barrier [31]. Therefore the antagonism of DA D2 receptors by DOM that leads to potentiation of GnRH action on LH release and induction of spawning is at the level of the pituitary [22,31]. Regardless, both formulations effectively induced spawning in *L. pipiens* in the breeding season. In only 4 days were able to harvest nearly 84,000 eggs from the 31 females that successfully laid eggs. Based on our results, we recommend the mixture of GnRH-A+MET over GnRH-A+DOM because of the ease of preparation and use.

We sought to further test GnRH-A+MET for out-of-season breeding under laboratory conditions. Firstly, we obtained only 3 fertilized egg masses from 7 females and 7 males collected in mid-November and injected with GnRH-A+MET in mid-December after being kept for approximately 1 month in the laboratory. The important innovation we report here is the improved out-of-season breeding outcomes. It is clear that a single injection of GnRH-A+MET works well with animals that are sexually mature in the natural breeding season. We reasoned that part of the initial low success out-of-season related to either reduced pituitary sensitivity to GnRH, and/or to reduced stores of gonadotropins in the pituitary. Both possibilities are well-documented in seasonally breeding poikilotherms, for example, in goldfish [32,33]. Therefore, we used the principle of GnRH priming to enhance the response of gonadotrophs in the anterior pituitary to subsequent GnRH treatment. It is well-known that GnRH can upregulate GnRH receptor numbers and LH production in the vertebrate pituitary by a mechanism called GnRH self-priming [15,34,35]. Therefore, in the present study we gave 10% of the GnRH-A dose (0.04 μg/g BW) in GnRH-A+MET as a priming dose to both males and females. This was followed 24h later by GnRH-A+MET, a mixture of the same GnRH-A and the DA antagonist MET. We report that 11/12 females laid eggs with a very high level of fertilization. The out-of-season percent fertilization estimates in the primed group were on average 85%, which is similar to fertilization rates (>90%) in leopard frogs caught and induced with GnRH-A+MET only in the natural breeding period. Additionally, in another study a priming injection of the GnRH agonist Leuprorelin (pHis-Trp-Ser-Tyr-D-Leu-Leu-Arg-Pro-NHEt) improved Leuprorelin-induced spermiation and ovulation in Gϋnther's toadlet, *Pseudophryne guentheri;* subsequent *in vitro* fertilization rates were very high [36]. These results indicates important advantages of GnRH priming in anurans.

The female *L. pipiens* that were induced to spawn in the natural breeding period and out-of-season had similar body sizes and similar egg mass weights. However, there was a difference in the number of eggs produced. The females in the natural breeding season produced on average ~2,764 eggs, whereas those out-of-season females produced only 1584 eggs. While these levels of egg production are within the normal but variable numbers for *L. pipiens* (800–7,500 per female [37]), there are several plausible explanations for this difference. The group of females caught in mid-November to early December 2011 may simply have had fewer developed eggs than those collected the previous spring in 2011. This could be natural variation from one cohort to another that would depend on nutritional conditions in the spring an summer following the breeding season. There were virtually no eggs remaining in the body cavity of those injected females that spawned, so partial release of the eggs cannot be the reason for differences in the number of eggs laid. Alternatively, it is possible that the relatively short period the out-of-season animals spend in cold-water conditions that simulated winter water temperatures was not enough to allow full ovarian development [38]. It will be important to determine the environmental and nutritional conditions to maximize egg numbers for out-of-season induced spawning in captive *L. pipiens.*

Another important consideration addressed in our study is the development of tadpoles obtained from captive breeding outside of the normal reproductive period. Generation of tadpoles for physiological and ecotoxicological experiments, and for captive breeding and reintroduction programs of endangered species all require large numbers of normal healthy tadpoles that grow and metamorphose. An additional requirement for reintroduction programs is the maintenance of genetic diversity, and therefore, careful management of broodstock and their offspring. We studied the 3 egg masses obtained from pairs injected only with GnRH-A+MET (masses 1–3) and 2 egg masses obtained from the GnRH-A priming protocol (masses 4–5). Levels of fertilization varied from 10–97.2%, but the variations in developmental parameters measured were not related to this fertility estimate. The number of days from fertilization to metamorphosis (DTM) varied from 85–118, which is well within the wide range (7–42 weeks) found in the literature [27,39-41]. Such variations reflect the extreme developmental plasticity and response to variable environmental conditions typical of anurans tadpoles [42]. Weight at metamorphosis was generally between 0.8 and 1.0 g, similar to previous reports for eggs obtained in the wild but raised outdoor in mesocosms [41], but somewhat smaller than what we previously reported for tadpoles obtained from captive breeding [27], reflecting potential genetic differences in the parents, and/or the different rearing conditions. Tadpoles from egg mass 5 were the only group that was obviously different from the others. Tadpoles from egg mass 5 metamorphosed quicker, despite having higher mortality, higher incidence of abnormalities and many fewer that actually reached metamorphosis. The higher mortality and abnormalities are likely related to the genetics of the parents but we cannot attribute this to a female-based or a male-based effect. We do not have any other data from these parents since they have only been bred once. The metamorphs from egg mass 5 had a higher condition factor relative to the other 4 cohorts. Their bigger size and increased condition factor in likely related to rearing conditions. We did not adjust tadpole densities during development to compensate for the different rates of mortality. Regardless, it is clear that there are differences between the breeding couples in captivity that reflect typical variations in natural populations.

## Conclusions

We provide evidence for successful captive breeding of the leopard frog, *L. pipiens.* This species may serve as a good model to use for the further development of robust spawning induction methods for endangered anurans because until now it has been considered very difficult to breed in captivity. The AMPHIPLEX method to inject both sexes simultaneously with a solution containing a GnRH agonist and a DA antagonist has been used successfully in 6 anuran species [27,43,44].

## Competing interests

The authors declare that they have no competing interests.

## Authors’ contributions

VLT developed the spawning induction method, designed, performed all breeding experiments with *L. pipiens* and wrote the paper. FS collected all *L. pipiens* and contributed to development of spawning conditions required. LNM and CKM designed and conducted the developmental studies. EB and BF contributed to the feeding, maintenance and hibernation protocols. AB performed some of the fertility studies. LT contributed to the design of the out-of-season breeding and tadpole maintenance protocols. All authors read and approved the final manuscript.

## Supplementary Material

Additional file 1: Figure S1 Tadpoles and froglets resulting from induced spawning out of the normal breeding season using the AMPHIPLEX method. Shown are photographs of *Lithobates pipiens* by Dr. A. Morin (Department of Biology, University of Ottawa). Click here for file
