# Supplementary material for: Efficient induction of spawning of Northern leopard frogs (Lithobates pipiens) during and outside the natural breeding season
Source: Reprod Biol Endocrinol. 2013 Feb 25;11:14. doi: 10.1186/1477-7827-11-14 (PMC3598769; doi:10.1186/1477-7827-11-14)
Supplement: Additional file 1: Figure S1 — Tadpoles and froglets resulting from induced spawning out of the normal breeding season using the AMPHIPLEX method. Shown are photographs of Lithobates pipiens by Dr. A. Morin (Department of Biology, University of Ottawa). [file 1477-7827-11-14-S1.pdf]

Supplemental figure 1.

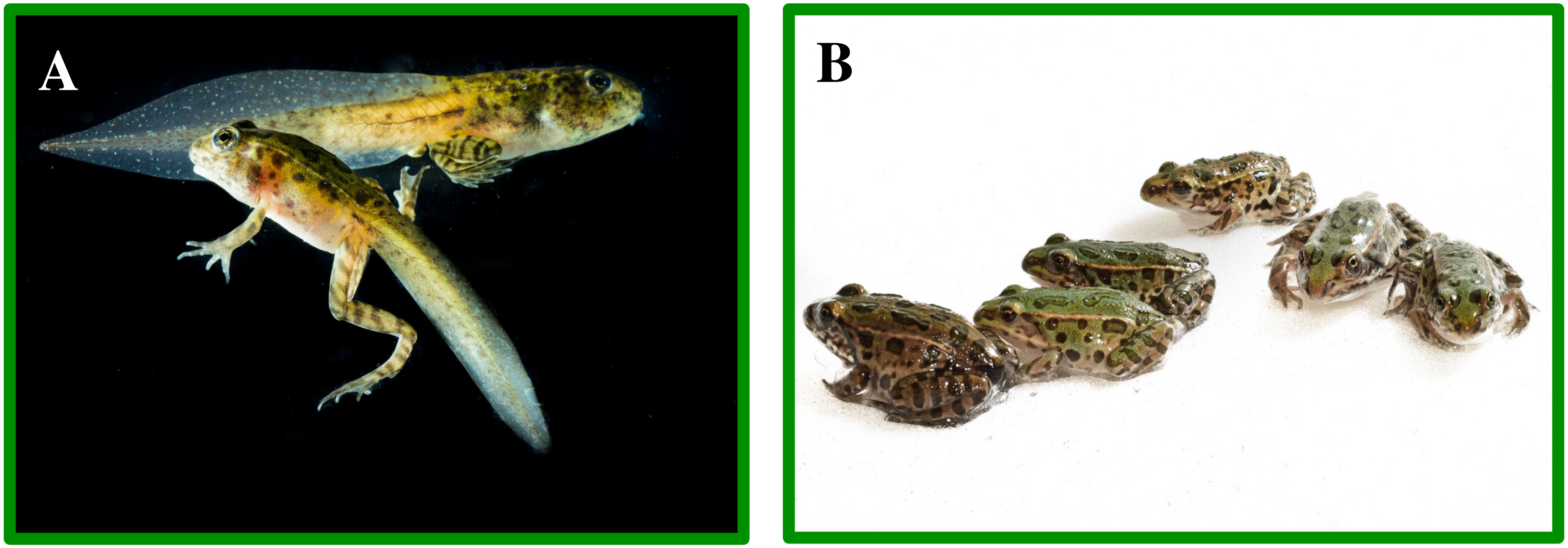

Lithobates pipiens **tadpoles (A) and metamorphs (B) from Trial 1 out-of-season breeding following spawning induction with AMPHIPLEX. Photos courtesy of Dr. Antoine Morin**
